# The role of lymphatics in intestinal inflammation

**DOI:** 10.1186/s41232-021-00175-6

**Published:** 2021-08-17

**Authors:** Ryota Hokari, Akira Tomioka

**Affiliations:** Department of Internal Medicine, National Defense Medical College, 3-2 Namiki, Tokorozawa, Saitama, 359-8513 Japan

**Keywords:** Lymphatic vasculature, Inflammation, Lipid absorption

## Abstract

The lymphatic vasculature returns filtered interstitial arterial fluid and tissue metabolites to the blood circulation. It also plays a major role in lipid absorption and immune cell trafficking. Lymphatic vascular defects have been revealed in inflammatory diseases, Crohn’s disease, obesity, cardiovascular disease, hypertension, atherosclerosis, and Alzheimer’s disease. In this review, we discuss lymphatic structure and function within the gut, such as dietary lipid absorption, the transport of antigens and immune cells to lymph nodes, peripheral tolerance, and lymphocyte migration from secondary lymphoid tissues to the lymphatics and the immune systems. We also discuss the potential roles of these lymphatics on the pathophysiology of inflammatory bowel disease and as new targets for therapeutic management.

## Background

The lymphatic system in the intestine has a specialized network of structures that transport tissue fluids, macromolecules, hormones, and lipids. It interacts closely with the immune system. Because the intestinal mucosa is constantly exposed to the commensal microbiota and lymphatic vessels (LVs) are highly permeable to bacteria, the lymphatic system plays a significant role in immunoregulation. Recent work suggests that disturbances in the lymphatic system are involved in Crohn’s disease (CD) pathogenesis and are a therapeutic target for this disease.

## Anatomy of the lymphatic vasculature

The LVs have at least four important functions: (1) tissue fluid homeostasis, (2) providing a route for nutrient distribution, especially lipids, (3) providing a route for immune cell trafficking from peripheral tissues into lymph nodes (LNs), and (4) transport of incretin hormones produced by neuroendocrine cells.

Blood plasma is continuously filtered from the arterial capillary into the interstitial space, where excess fluid cannot be fully reabsorbed by venous capillaries and taken up by the initial lymphatics [1]. Initial lymphatics lack a continuous basement membrane and perivascular mural cells. Lymphatic endothelial cells (LECs) within the initial lymphatics are interconnected through discontinuous button-like junctions and facilitate interstitial fluid and macromolecule uptake [2]. The collecting lymphatics display a continuous zipper-like junction located at the cell borders with no openings. The collecting vessels are less permeable because of the continuous cell-cell junctions, and small intestinal villi contain a blood capillary network and central LVs, termed lacteals. Lacteals have the same structure as the initial lymphatics and consist of a non-fenestrated single layer of LEC without a basement membrane [3]. Although lacteals are absent in the colon, a network of lymphatics is observed on the luminal surface of the lamina propria and in the base of the lamina propria. It has an extensive submucosal network.

## The role of lymphatics in lipid absorption

The major factor controlling the intestinal absorption of materials is their solubility. Water-soluble substances (amino acids, glucose, short-chain fatty acids) are transferred to the portal vein through blood capillaries, while lipophilic substances associated with chylomicrons enter lymphatics [4]. Digestion and absorption of dietary lipids in the intestinal tract involves multiple steps. In the first step, neutral lipids are hydrolyzed into fatty acids and monoglycerides in the gut lumen, leading to transfer through the apical membrane of enterocytes, where endoplasmic reticulum enzymes repackage them into triglycerides (TGs). In the second step, the TGs are assembled into chylomicrons (CMs) with phospholipids, cholesteryl esters, cholesterol, and ApoB. And then they are released from the basolateral membranes of enterocytes [5]. Dietary lipid transport by the lymphatic system is generally considered to involve passive unregulated drainage. However, numerous studies have shown that active processes tightly regulate lipid transport by lacteals. CM uptake by lacteals is regulated by the transcription factor pleomorphic adenoma gene-like 2 (PlagL2). PlagL2 null mice have functional enterocytes that secrete CMs, but CMs fail to enter the lacteals [6]. A recent study showed that VEGF-A signaling regulates CM uptake through the modulation of lacteal junctions [7]. High levels of VEGF-A induce button-to-zipper junction transformation in the lacteals, which inhibits CM entry into the lymphatic capillaries, causing lipid malabsorption and reduced weight gain associated with a high-fat diet. It causes an opposite effect on blood vessels, increasing blood capillary leakage by opening normally closed cell-cell junctions.

## Epidemiology of CD and lipid consumption

CD is a chronic inflammatory bowel disease that frequently occurs in the small intestine and colon [8]. Although primarily genetic in origin, immunological and environmental factors have been proposed, and the underlying mechanism remains unclear. Corticosteroids are considered a major therapeutic option for inducing remission in patients with active CD [9]. However, the side effects of corticosteroids, such as Cushing’s appearance, bone demineralization, and increased rate of infection, can be harmful. The incidence of CD is related to dietary intake of total fat, suggesting that CD is a lifestyle-related disease [10]. Four meta-analyses compared the efficacy of inducing remission of active CD patients between enteral nutrition and corticosteroids, showing the pathophysiology of diet in CD [11–14]. In 2014, the European Society of Pediatric Gastroenterology, Hepatology, and Nutrition (ESPGHAN) and the European Crohn’s and Colitis Organization (ECCO) issued revised consensus guidelines recommending that enteral nutrition be considered as the first-line induction therapy for children with CD [15]. A randomized, controlled study showed that the addition of fat to enteral nutrition decreased its therapeutic effect against active CD, suggesting that dietary fat influences intestinal inflammation [16].

Considerable efforts have been directed at defining the influence of nutrition on the immune system. There is considerable evidence implicating dietary fat as a modulator of different immune functions. The dependence of lymphocyte function on lipid absorption in intestinal lymphatics has been proposed. Using intravital microscopy, we have shown that the administration of lipids increases the flux of lymphocytes from the intestinal mucosa to the mesenteric lymph node (mLN) through the intestinal lymph [17]. The contraction frequency of intestinal collecting lymphatics was also enhanced by olive oil administration.

## Lymphatics and the immune system

Because the initial LVs are greatly penetrable to objects below 1 μm, viruses and bacteria are able to infiltrate the LVs. Afferent LVs are well-known as the channels which immune cells and antigens are carried to the draining LNs for immune responses. Tissue-resident dendritic cells (DCs) migrate to the draining LNs via afferent lymphatics to carry antigens. Tissue-derived antigens are afterwards processed by lymph node-resident antigen-presenting cells [18]. A DC can infiltrate a LV through gaps in the perilymphatic basement membrane [19].

The purpose of LNs is to filter the lymph from the lymph’s draining area and detect antigens [20]. Immune reactions or immunosuppression occurs in the LNs. LNs are surrounded in a primarily collagen capsule lined with lymphatic endothelial cells which form subcapsular sinuses. Shortly after naïve lymphocytes enter a LN, they proceed along stromal networks to scan the surface of antigen-presenting cells for cognate antigens. LN contains specialized stromal cells including LECs, blood endothelial cells, follicular dendritic cells (FDCs), marginal reticular cells (MRCs), integrin α7^+^ pericytes (IAPs), and fibroblastic reticular cells (FRCs). LN structure is subdivided into 3 parts, such as cortical, paracortical, and medullary areas according to the distribution of specific stromal cells. FDCs resides cortex and produces the chemokines which attract B cells. FRCs resides in the paracortical area and have a key function in T cell homeostasis [21, 22].

Recent in vivo live imaging studies have shown that stromal cells play a crucial role during lymphocytic migration within LNs. Naïve lymphocytes form intimate interactions with FRC networks [23, 24]. Following transendothelial migration from the bloodstream by high endothelia1l venules (HEVs), naive lymphocytes access FRC networks at specific sites and actively inch along the surface of FRC networks. Stromal cells coordinate with lymphocytes and DCs, supply scaffolds on which these cells migrate, and recruit them into niches by secreting chemokines. Macrophages exist in lymphatic endothelial cells located in the subcapsular sinuses and medullary sinuses. Subcapsular sinuses macrophages rapidly capture lymph-borne pathogens, thus preventing their systemic dissemination [25]. The medullary sinuses are additionally covered by lymphatic endothelial cells.

In MLNs, DCs dictate the type of helper T responses toward Th1, Th2, Th17, or regulatory T (Treg)cells. DCs also induces gut-tropic adhesion molecules such as α4β7 integrin on T cells. The immune mechanisms that govern UC and CD disease process include the recruitment of pathogenic Th17 cells to the intestinal mucosa. A recent study showed that Th17 cells in MLNs from CD patients were characterized by a predominant Th17 population displaying a pathogenic/cytotoxic gene signature (IL23R, IL18RAP, and GZMB, CD160, PRF1, while non-pathogenic/regulatory genes (IL9, FOXP3, CTLA4) were more elevated in UC [26].

## The role of LECs in peripheral tolerance

Oral tolerance is not able to be inducible without mLN [27]. Compiling evidence reveals that LECs, stromal cells, CD103^+^ DCs drained from an induction site, and regulatory T cells are associated in this mechanism. LECs surround lymph node sinuses and are proposed to play a part in peripheral tolerance instead of T cell activation. This tolerogenic potential is restricted only to LECs in the LNs, especially those distributed in the medulla. LN LECs are able to present antigens to immune cells directly [28, 29]. LN LECs can sample exogenous antigens efficiently through the high phagocytic and endocytic capacities. Both MHC class I and MHC class II molecules are present in LN LECs. A virtual lack of co-stimulatory molecules was also detected in LN LECs. The representation of lymphatic endothelial cells in the medullary sinus expressing high-level programmed cell death 1 ligand 1 (PD-L1) is controlled by lymphotoxin-β receptor signaling and B cells [30].

LECs greatly express peripheral tissue antigens but are not the case in tissue lymphatics [31]. LECs express MHC-I and MHC-II (omitting costimulatory molecules) and display the MHC-I antigen through direct and cross-presentation. Direct presentation of peripheral tissue antigens (PTAs) can cause abortive proliferation and deletion, on account of both a lack of co-stimulation and active PD-L1 engagement. LN LECs also express immunosuppressive enzymes, such as indoleamine dioxygenase and inducible nitric oxide synthase, which inhibit dendritic cell maturation. Single-cell RNA sequencing has shown that up to six types of LECs exist in human LNs from axillary, head, and neck, corresponding to distinct locations in the node, with each expressing distinct chemokine profiles and decoy receptors that scavenge chemokines to maintain gradients of chemotactic signals that guide immune cells to areas of their highest functional concentration [32].

## Sphingosine-1 phosphate’s (S1P) effects on lymphocyte migration from secondary lymphoid tissue sites

Naïve lymphocytes that do not come into contact with their target antigen exit the LNs via efferent lymphatics. It is commonly known that S1P is an essential regulatory molecule when lymphocytes migrate from secondary lymphoid organs (SLOs) including but not limited to LNs, or Peyer’s patches (PPs) [33]. S1P mediates its functions through S1PRs(S1P_1_–S1P_5_). In blood, S1P predominantly arises from red blood cells and platelets, and its concentration is kept rather high (several hundred nanomoles per liter). Conversely, S1P concentration in lymphoid tissues is sustained at a reduced (nanomolar) level by the action of S1P lyase and dephosphorylation enzymes [34]. This variation results in a concentration gradient in the S1P.

Naïve lymphocytes heavily convey the receptors for S1P (S1P-R). Once naïve lymphocytes migrate into secondary lymphoid tissues due to the influence of chemokines and they do not find any antigens, they will then emigrate from the secondary lymphoid tissues into lymphatics as a result from the high concentration of S1P in the lymph fluid. Oppositely, after antigens have been exhibited to naïve T lymphocytes, the expression of S1P-R on T lymphocytes is lowered [35]. The departure of T lymphocytes from secondary lymphoid follicles is restrained, and they are kept inside lymphoid tissues. One physiological mechanism for removing S1P_1_ from the T cell surface is binding to the activation antigen CD69, which prompts the internalization and degradation of S1P_1_ [36]. Hence, antigen-specific T lymphocytes acquire activation signals from antigens, and accessory stimulatory molecules replenish S1P-R expression on their surfaces. T lymphocytes are carried to the periphery again in an S1P-dependent manner.

S1PRs are involved in the physiological migration of lymphocytes and the migration of pathological cells. It is believed that S1PRs contribute in intestinal food allergies and inflammatory bowel diseases [37]. S1P secretion is adjusted by α9 integrin and its ligand, tenascin-C, colocalized in the medullary and cortical sinuses of draining LNs acting as a gate lymphocyte exit under inflammatory conditions [38]. Blockade of α9 integrin-mediated signaling decreases lymphocyte egress from draining LNs in several experimental models, as well as experimental autoimmune encephalomyelitis.

During inflammation, lymph flow escalates to restrict edema and prevent antigen cell transport. In this case, proinflammatory cytokines increase expressions of adhesion molecules, such as P-selectin, E-selectin, and L-selectin ligand, on HEVs, aiding the access of lymphocytes into LNs [39]. In such circumstances, lymphocyte egress from LNs decreases too [40]. This decrease is regulated by the downregulation of S1P-R1 expression in lymphocytes. Therefore, the escalated lymph flow and the decreased egress of lymphocytes increase lymphocyte accumulation in LNs. This accumulation causes the increased chance for lymphocytes to encounter cognate increases therefore enhancing immune functions.

S1P also assists in maintaining the integrity of the HEVs. For example, a research study conducted on mice that lacked podoplanin (PDPN) lost HEV integrity and developed spontaneous bleeding in their LNs after immunization. PDPN manifested on FRCs surrounding HEVs operates as an activating ligand for platelet CLEC-2. PDPN-CLEC2-mediated platelet activation results in local SIP release from platelets, leading to maintenance of vascular integrity in HEVs [41].

## Histopathological features of inflammatory bowel disease (IBD)

The pathological features observed in CD patients are interstitial edema, mucosal exudates, and extensive dilation of lacteals. Hyperpermeability of blood vessels increases interstitial fluid accumulation under inflammatory conditions, resulting in increased lymph flow to prevent the development of edema. Decreases in lymph transport during chronic inflammation have also been reported [42]. Lymph flow decreased within days in the murine model of intestinal inflammation and murine models of acute and chronic skin inflammation. Conversely, lymph flow increases during recovery in acute intestinal and skin inflammation models but not in chronic skin inflammation models [43–45].

Blood vessel densities in the colon during active CD are significantly increased in the intestinal mucosa and submucosa, particularly during the acute phases of inflammation [46]. However, in the colon, lymphatics are normally restricted to the region below the muscularis mucosa, with only occasional extensions through the muscularis to the base of the colon, suggesting that a relative decrease in lymphatics leads to increased tissue fluid [47]. We also measured vessel density in CD patients according to pathological activity. Blood vessel densities were higher in actively inflamed mucosa and showed a positive relationship with disease activity. In contrast, lymphatic vessel densities were higher in quiescent sections of CD patients than in controls, but lymphatic vessel densities of active sections were not higher than quiescent sections, suggesting the presence of damaged active lymphatic CD mucosa [48]. Clinically, lower LV density was associated with a higher risk of endoscopic disease recurrence after surgery, suggesting that patients could benefit from improved lymphatic flow [49, 50].

Pathologically, LV obstruction and dysfunction are long-recognized features observed in CD [51, 52]. Obstructive lymphoid aggregates, which contain lymphocytes and macrophages were major histopathological features described in CD and can result from poor lymphatic drainage, which reflects impaired lymphatic pumping and conduction [53]. These defective lymphatics might be unable to promote efficient drainage and inflammation resolution.

Lymphoid aggregates, inflammatory granulomas, and “fat wrapping” (fat creeping) suggest a general failure of lymphatic transport of lipids and inflammatory cells [54]. It was recently proposed that fatty acids released from adipocytes within creeping fat shift the metabolic state of muscle cells directly in the intestinal muscularis externa to drive muscle proliferation and fibrosis, resulting strictures [55]. Given the role of inflammation in adipose tissue expansion and the adipogenic properties of the lymph, chronic lymph leakage in CD may contribute to the generation of creeping fat. In turn, increased visceral adiposity may sustain chronic inflammation.

In an animal model of intestinal ileitis, impaired contractile function of mesenteric collecting lymphatics was observed [56, 57]. Blocking lymphangiogenesis with anti-VEGFR3 antibody treatment in an animal model of IBD aggravates inflammation and submucosal edema and increases leukocyte infiltration [58, 59]. In contrast, resolution of chronic inflammation may require restoration of proper lymphatic function, removing inflammatory cells, mediators, and bacterial antigens from inflamed sites. Systemic treatment with VEGF-C significantly reduced the clinical and histological signs of inflammation, expanded the lymphatic vascular network, reduced the density of immune cells, and decreased inflammatory cytokine expression in the inflamed colon [60].

## Therapeutics targeting lymphatic function improvement

Cyclooxygenase (cyclooxygenase 1 and 2) or inducible nitric oxide synthase inhibitors improve lymphatic pumping in non-contracting lymphatics in experimental IBD, suggesting that prostanoids and nitric oxide released during intestinal inflammation contribute to diminished lymphatic contractile activity [61, 62]. Tacrolimus reduces lymphedema without immunosuppression by increasing the formation of collateral lymphatic vessels [63]. Y-27632 induces intestinal lacteal junction “zippering” and conversion of “button-like” cell-cell junctions to linear junctions [7]. Exercise improves lymphatic contractile function [64].

IBD is also linked with platelet hyperactivation and coagulation, inducing venous thrombosis and congestion, including mesenteric venous thrombosis [65, 66]. Recently, it was reported that the binding of activated platelets to PDPN on LECs induced thrombus formation in the LV [67, 68]. In addition, platelets inhibit the proliferation of human LECs [69]. In a murine colitis model, induction of colitis induced the migration of platelets into the lymphatic system. Platelet depletion by anti-platelet antibody treatment significantly reduced the clinical and histological signs of inflammation, expanded the lymphatic vascular network, reduced the density of immune cells, and decreased the expression of inflammatory cytokines in the inflamed murine colon [48]. Anti-platelet therapy is also a candidate for novel therapy by improving lymphatic function.

## Conclusions

The lymphatic vasculature plays a major role in transporting antigens, immune cell trafficking, and lipid absorption. The lymphatic vasculature in the inflamed mucosa controls inflammation through the clearance of inflammatory cells. LECs in LNs are involved in peripheral tolerance rather than T cell activation. In CD, impaired lymphatic conduction and pumping may be involved in the pathophysiology of chronic refractory disease. Overall, these results reveal that therapeutics targeting lymphatic function improvement are a novel and promising approach for treating intestinal inflammatory diseases, including IBD.

## Data Availability

Data sharing is not applicable to this article as no datasets were generated or analyzed during the current study.

## Abbreviations

CM: Chylomicrons
TGs: Triglycerides
CD: Crohn’s disease
LECs: Lymphatic endothelial cell
ApoB: Apolipoprotein B
DCs: Dendritic cells
LT: Lymphotoxin
PDPN: Podoplanin
FRCs: Fibroblastic reticular cells
HEVs: High endothelial venules
CLEC-2: C-type lectin-like receptor 2
LVs: Lymphatic vessels
